# Capsaicin and Quercitrin Maintained Lipid Homeostasis of Hyperlipidemic Mice: Serum Metabolomics and Signaling Pathways

**DOI:** 10.3390/foods13233727

**Published:** 2024-11-21

**Authors:** Yanxia Wu, Weihua Liu, Rongrong Wang, Yunhe Lian, Xinying Cheng, Ruili Yang, Xianghong Wang, Si Mi

**Affiliations:** 1 College of Food Science and Technology, Hebei Agricultural University, No. 2596 Lekai South Street, Baoding 071000, China; yanxiawuhebau@163.com (Y.W.); weihualiuhebau@163.com (W.L.); rongrongwanghebau@163.com (R.W.); wangxianghonghebau@163.com (X.W.); 2 Chenguang Biotech Group Co., Ltd., Handan 057250, China; 18561166392@163.com; 3 Hebei Chenguang Testing Technical Services Co., Ltd., Handan 057250, China; xinyingchengcg@163.com (X.C.); ruiliyangcg@163.com (R.Y.)

**Keywords:** hypolipidemic effect, capsaicin, quercitrin, metabolomics, signaling pathways

## Abstract

Capsaicin and quercitrin have proved to be two major ingredients in fresh chili pepper. However, the effect of these two compounds on hyperlipidemia and the related molecular mechanisms were still unclear. This work was performed to examine the hypolipidemic capacity of capsaicin and quercitrin as well as the related signaling pathways. Hyperlipidemia was induced in mice by feeding them with a high-fat diet for 4 weeks. Both capsaicin and quercitrin were beneficial to inhibit a rise in fasting glucose, total cholesterol, total triglycerides, low-density lipoprotein cholesterol, and total bile acids and to lift the level of high-density lipoprotein cholesterol in the serum. The optimal lipid-lowering data were achieved in the capsaicin and quercitrin/3:1 group. Supplementation with capsaicin and quercitrin both singly and together in the feed caused a significant influence on the metabolite profiles of mouse serum. The signaling pathway for the hypolipidemic effect of capsaicin and quercitrin was related to the down-regulation of epidermal growth factor receptor (EGFR) but the up-regulation of phosphatidylin-ositol-3-kinase (PI3K), protein kinase Bb(Akt), farnesoid X receptor 1 (FXR1), and cholesterol 7α-hydroxylase (CYP7A1). This study confirmed the jointly hypolipidemic effect of capsaicin and quercitrin, which would benefit the valorization of chili pepper resources.

## 1. Introduction

Chili pepper is one of the most popular vegetable crops worldwide. According to the statistics, the global production of chili pepper is approximately 20 million tons per year, and the market size has reached USD 9249.90 million [1]. Chili peppers, classified into various varieties, can be utilized for multiple purposes. They can be consumed as fresh vegetables or processed as raw materials [2,3]. In recent years, the enhancement of body health via consuming chili peppers has received widespread attention from both consumers and the academic community.

There was a survey performed on the daily diet of 500,000 Chinese people aged 35–79 years old and from 10 different areas [4]. The meta-analysis data indicated that people who ate spicy food had an overall lower mortality risk as well as a lower probability of dying from cancer and heart and respiratory diseases compared to those who did not frequently eat spicy food [4]. Almost all available reports have attributed the functional activity of chili pepper to the capsaicinoids, which proved to exhibit anti-obesity, antioxidant, anti-cancer, and anti-inflammation bioactivities [1,3,5]. A team led by scholars from the Cleveland Clinic conducted statistical analyses on the medical and dietary data of over 570,000 individuals from Iran, China, Italy, and the United States [6]. The results showed that compared to those who seldom or never consumed chili peppers, people who regularly consumed chili pepper exhibited a 26% decline in cardiovascular disease mortality [6]. These findings imply that chili pepper may possess the potential to regulate lipid metabolism.

A large body of evidence has revealed that dietary capsaicin was effective to alleviate hyperlipidemia symptoms in different experimental animals fed with a high-fat diet [7,8,9,10,11]. Although the lipid-lowering activity of capsaicin has been confirmed, the involved molecular pathway has not been fully elucidated. Moreover, it has been well accepted that chili pepper is rich in multiple ingredients like vitamin C, flavonoids (e.g., rutin, luteolin, ferulic acid), and minerals (e.g., Ca, Fe, Zn, Mn) in addition to capsainoids [1,3]. Therefore, we speculate that when consumers eat chili pepper as a whole fruit, it is not just the capsaicin that plays a hypolipidemic role. The simultaneous involvement of other bio-active components might also lead to lipid-lowering performance. Our previous studies found that quercitrin was a dominant flavonoid compound present in the chili pepper fruit, being 108.07 μg/g [2]. Taking all of the above into account, we conducted this study to evaluate the performance of capsaicin and quercitrin (Supplementary Figure S1) both singly and in combination on the improvement of hyperlipidemic mice created with a high-fat diet. The underlying mechanism would be unraveled via metabolite profiling in the animal serum and the expression levels of key signaling proteins.

## 2. Materials and Methods

### 2.1. Chemicals, Standards, and Assay Kits

Quercitrin with >98% purity was booked from Alpha Biotechnology Co., Ltd. (Chengdu, China). Standards for capsaicin (>97% purity) and simvastatin (>98% purity) were ordered by McLean Biochemical Technology Co., Ltd. (Shanghai, China). LC/MS-grade acetonitrile and LC-grade methanol was obtained from Thermo Fisher Scientific (Shanghai, China). Commercial assay kits for the quantification of both high- and low-density lipoprotein cholesterol, total cholesterols, total bile acids, BCA protein, and total triglycerides were supplied by Jiancheng Bioengineering Institute (Nanjing, China). An Omni-EasyTM one-step PAGE gel rapid preparation kit was provided by Shanghai Yazyme Biotechnology Co., Ltd. (Shanghai, China). CYP7A1 (CAS#YM1026, 58 kDa) was purchased from ImmunoWay Biotechnology Company (Suzhou, JS, China). EGFR (CAS#ab52894, 134 kDa) and FXR1 (CAS#ab129089, 70–75 kDa) were obtained from Abcam (Cambridge, United Kingdom). PI3K (CAS#4257, 85 kDa), P-Akt (CAS#4060, 60 kDa), and Akt (CAS#2638, 60 kDa) were ordered from Cell Signaling Technology (Danvers, MA, USA). GAPDH (CAS#10494-1-AP, 36 kDa), HRP-conjugated Affinipure Goat Anti-Rabbit IgG (CAS#00001-1), and Goat Anti-Mouse IgG (CAS#00001-1) were provided by Proteintech Group, Inc. (Wuhan, China).

### 2.2. Animals, Experimental Flow, and Grouping

All of the operations performed on the mice of this study were ethically licensed by the Animal Care and Use Committee of Hebei Agricultural University (No. 2022126).

Sixty C57BL/6J male mice (6 weeks old, average weight 18 ± 2 g, license number: SCXK (JING) 019-0010) were provided by Spafford Biotechnology Ltd. (Beijing, China). After coming to the lab, they were kept in a well-ventilated environment with 12 h of alternating light and dark. The room temperature was stabilized at 22 ± 2 °C, whilst the humidity condition was controlled in a range of 45%~60%. In this study, all experimental animals had free availability of water and food. Ten mice were chosen to organize the normal control (CK) group after a week of acclimation. These ten animals were fed with a basal diet (F002, Beijing Sparkford Biotechnology Co., Ltd., Beijing, China). The remaining 50 mice were given a diet with high fat content (SFD018, Beijing Spafford Biotechnology Co., Ltd., Beijing, China), which consisted of 58.6% basal diet, 15% lard, 20% sucrose, 5% casein, 1.2% cholesterol, and 0.2% sodium cholate.

The feasibility of the established model was assessed by comparing the levels of total cholesterol (TC) and triglycerides (TG) between the model (HLP) and CK groups. The animal model was considered workable if the TC and TG levels detected in the HLP mice were significantly higher (*p* < 0.05) than that of the CK mice. The hyperlipidemic animals were assigned into five groups at random, including model (HLP), simvastatin (administration with simvastatin at 10 mg kg^−1^ body weight, SIM), capsaicin (administration with capsaicin at 9 mg kg^−1^ body weight, Cap), quercitrin (administration with quercitrin at 3 mg kg^−1^ body weight, Quer), and capsaicin and quercitrin/3:1 (administration with a combination of capsaicin and quercitrin at 9 mg kg^−1^ body weight and 3 mg kg^−1^ body weight, Cap+Quer/3:1). Regarding the CK and HLP groups, the mice were intragastrically administered the same amount of 1% sodium carboxymethyl cellulose suspension solution, and the gavage volume was 0.1 mL/10 g body weight.

### 2.3. Determination of Oral Glucose Tolerance of Mice

An oral glucose tolerance test (OGTT) was conducted on the mice after intervention with capsaicin and quercitrin for 36 days with reference to the protocols established by Mi et al. [3]. After fasting for 12 h, the animals were orally administrated glucose at 2 mg g^−1^ body weight. The blood glucose (BG) level of the mice was examined using a Sinocare blood glucose meter after intervention for 0, 30, 60, and 120 min. The area under the glucose tolerance curve (AUC-OGTT) was calculated as follows:

AUC-OGTT = 0.5 × (BG at 0 min + BG at 30 min)/2 + 0.5 × (BG at 30 min + BG at 60 min)/2 + 1 × (BG at 60 min + BG at 120 min)/2

### 2.4. Measurement of Various Lipid Indicators in the Serum of Mice

When the administration finished, the water supply for all experimental animals were blocked for 12 h. Next, the blood of the mice was obtained from the eyeballs using a periorbital blood collection method, which was followed by cervical dislocation. The obtained blood samples were held at room temperature for half an hour and then subjected to a 10 min centrifugation at 3000 rpm and 4 °C. The supernatant was gathered and kept for later use at −80 °C.

The concentrations of total cholesterol (TC) and total triglycerides (TG) in the serum was assessed in compliance with the instructions developed by the assay kit manufacturer. As for TC analysis, the blank, calibration, and sample wells were filled with distilled water, cholesterol standard, and serum sample (each for 2.5 µL), respectively. Subsequently, 250 µL of the working solution was supplemented to each well, combined thoroughly, and maintained at 37 °C for 10 min. A wavelength of 500 nm was sent to record the absorbance of each well. For TG analysis, the cholesterol standard was replaced by the triglyceride standard, whilst the working solution was replaced by that required for triglycerides. The analytical procedures were similar to that of TC.

The contents of low- and high-density lipoprotein cholesterols were assessed following the instructions of the commercial test kits. In short, an equal amount of 2.5 µL distilled water, standard, and serum sample was added to three separated wells, followed by mixing with 180 µL of reagent 1. Then, the plate was maintained at 37 °C for 5 min, and the absorbance (A1) was measured at a wavelength of 600 nm. Followingly, 60 µL of reagent 2 was supplemented and the obtained solution was kept at 37 °C for another 5 min prior to recording the absorbance (A2).

The content of total bile acids (TBA) in the serum was examined in compliance with the test kit’s instructions. An equal amount of 3 µL of distilled water, standard, and serum sample was added to three separated wells, followed by mixing with 180 µL of reagent 1. Following a 5 min incubation at 37 °C, 60 µL of reagent 2 was added and reacted for one minute, and the absorbance value A0 was recorded at 405 nm. Three minutes later, the absorbance value A1 was measured to calculate the TBA content.

### 2.5. Metabolomics Analysis of Mouse Serum by UHPLC-Q-TOF/MS

After melting at 4 °C, a volume of 100 µL of serum sample was added to 400 µL of a cold solvent consisting of methanol and acetonitrile (1:1, *v*/*v*). The mixed solution was subjected to centrifugation to obtain the supernatant, which was then dried under a vacuum. Followingly, the dried sample was re-dissolved with 100 µL of water and acetonitrile (1:1, *v*/*v*) and then centrifuged to obtain the supernatant for the instrumental analysis.

The metabolite composition of mouse serum was evaluated by using an Agilent 1290 Infinity Ultra-High-Performance Liquid Chromatography (Thermo Scientific, Waltham, MA, USA) instrument coupled to a Triple TOF 6600 mass spectrometer (AB SCIEX, Boston, MA, USA). The operation details were in accordance to those published by our group [2]. A volume of 2 µL of sample was injected and eluted through an ACQUITY UPLC BEH Amide column (100 mm × 2.1 mm i.d., 1.7 µm in particle size) along with a mobile phase comprising (A) water with 25 mM ammonium acetate and ammonium hydroxide and (B) acetonitrile. The mobile phase flew through the column under a gradient program as follows: 95% B for 0.5 min, dropped linearly to 65% B within 6.5 min, further declined to 40% B within 1 min, held for 1 min, then climbed to 95% B within 0.1 min and maintained for 2.9 min. The LC eluent was converted into ions through electrospray ionization (ESI) under both positive and negative modes. The raw MS data were converted to MzXML files using ProteoWizard MSConvert (https://proteowizard.sourceforge.io/), and these files were then imported into the XCMS program (www.bioconductor.org/) for data processing, including raw peak extraction, data baseline filtration and calibration, peak comparison, inverse convolution analysis, peak identification, and peak area integration.

### 2.6. Determination of the Relative Expression Levels of Key Proteins by Western Blotting

Western blotting on the selected key proteins was conducted with reference to our published method procedures [3]. The mice liver tissues (100 mg) were put on ice for thawing and ground thoroughly with 1 mL of RIPA cracking solution (SEVEN, Beijing, China) comprising 1% PMSF (SEVEN, Beijing, China), protease, and phosphatase inhibitors (MCE, Los Angeles, CA, USA). Then, the sample solution was put on ice and held for 20 min, which was followed by a 20 min centrifugation (14,000× *g* and 4 °C) to obtain the supernatant. The crude protein amount of each animal group was achieved by using the commercial test kit (Nanjing, China). Briefly, the supernatant sample was combined with 5 × loading buffer, heated for boiling, and kept for 5 min. After cooling to the ambient temperature, the resulting sample buffer solution was mixed with 10% sodium dodecyl sulfate-polyacrylamide gel (SDS-PAGE) for one-hour electrophoresis. After that, a Bio-Rad semi-dry transfer instrument was applied to transfer the isolated fraction to the nitrocellulose membrane, which was subsequently submerged in a sequence of 5% skimmed milk powder and primary antibody for two hours and stored at 4 °C overnight. Subsequently, the nitrocellulose membrane was transferred to be immersed in a specific secondary antibody and kept at ambient temperature for one hour. The ultrasensitive chemiluminescence substrates (NCM Biotechnology, Sudan, China) were enhanced, and the protein bands were observed via a JUNYI gel imaging system (JY04S-3E, Beijing, China). Protein bands were analyzed by Image J software (1.52v, NIH, Bethesda, MD, USA).

### 2.7. Data Processing and Statistics

All the results of the current study are reported as mean values ± standard deviation (SD). Microsoft excel software (version 2021) with XLSTAT version 2023 as an add-in was used to conduct statistical data processing. Blank filtering was applied to remove ion peaks with missing values > 50%. After blank filling and KNN filling, data filtering was performed to remove the features with RSD > 50%. R software version 3.6.3 was used to perform multivariate statistics on the processed data. A *p* value obtained from one-way ANOVA < 0.05 was defined to have statistical significance. Figures were prepared using GraphPad Prism version 7.0. The pathways of differential metabolites were elucidated based on the Kyoto Encyclopedia of Genes and Genomes (KEGG) (http://www.genome.ad.jp/kegg/ (accessed on 2 March 2024)) using KAAS (KEGG Automatic Annotation Server) database.

## 3. Results and Discussion

### 3.1. Variations in Lipid Indicators in the Serum from Different Groups of Mice

Images of mice from the six different groups are shown in Supplementary Figure S2. The mice from the normal control (CK) group had shiny hair and moved flexibly, whereas those from the HLP group had dull hair, slow movement, severe hair loss, and preferred to hide in corners. Compared with the HLP group, the animals with drug intervention showed certain improvements in hair glossiness, movement flexibility, and hair loss.

**Figure 1:**
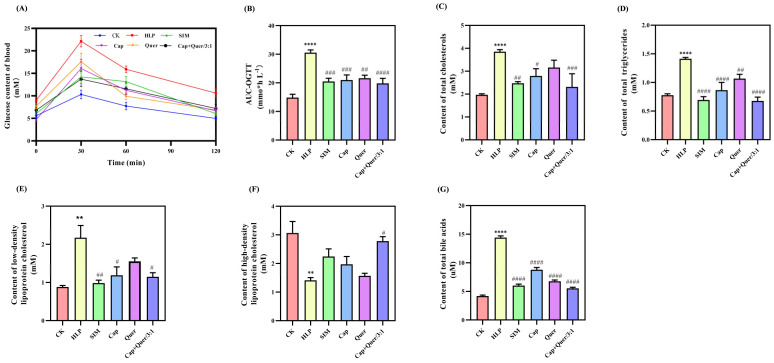
The effect of capsaicin, quercitrin, and their combination on the (**A**) glucose content in the blood, (**B**) area under the curve of oral glucose tolerance test (AUC-OGTT), (**C**) content of total cholesterol, (**D**) content of total triglycerides, (**E**) content of low-density lipoprotein cholesterol, (**F**) content of high-density lipoprotein cholesterol, and (**G**) content of total bile acids. CK, normal control; HLP, hyperlipidemia; SIM, simvastatin; Cap, capsaicin; Quer, quercitrin; Cap+Quer/3:1, capsaicin and quercitrin at a ratio of 3:1. Compared to the control group, ** *p* < 0.01 and **** *p* < 0.0001. Compared to the hyperlipidemic group, # *p* < 0.05, ## *p* < 0.01, ### *p* < 0.001, and #### *p* < 0.0001.

Lipid and glucose metabolism disorders usually occur concomitantly [12]. Evidence has revealed that the prevalence of glucose metabolism disorder in the hyperlipidemia cohort is 1.7 times greater than that in normal people [13]. This implies that hyperlipidemia lifts the risk of other related metabolic syndromes. The oral glucose tolerance test (OGTT) performed in this study confirmed the previous findings that a long-term high-fat diet was often accompanied by poor glucose tolerance. As shown in Figure 1A, all mice showed a generally similar trend in the fasting blood glucose (FBG) value, being a steady climb within the first 30 min and then a dynamic decline until 120 min. The highest FBG content was always observed in the HLP mice, whilst the lowest value was present in the CK group (Figure 1A). Administration with capsaicin and quercitrin eased the rise in FBG levels in the HLP animals. The area under the curve of the oral glucose tolerance test (AUC-OGTT) was applied to evaluate the capacity of mice to modulate or tolerate blood glucose. A remarkably larger AUC-OGTT value (30.54 ± 2.78 mmol·h/L) was achieved from the HLP animals compared to that (14.85 ± 3.70 mmol·h/L) of the CK mice (Figure 1B). As for the mice fed with different combinations of capsaicin and quercitrin, significantly (*p* < 0.05) smaller AUC-OGTT values were achieved than those of the HLP group (Figure 1B). Moreover, the lowest value of 19.81 ± 4.68 mmol·h/L was found in the Cap+Quer/3:1 group, indicating the best hypoglycemic impact. A similar effect was reported by our group on a combination of capsaicin and quercetin in T2DM mice [3]. The current data revealed an enhanced interaction of capsaicin and quercitrin, especially at a ratio of 3:1, to regulate glucose metabolism in HLP mice.

Hyperlipidemia features lifted concentrations of total cholesterol (TC), total triglycerides (TG), and low-density lipoprotein cholesterol (LDL-C) but a declined concentration of high-density lipoprotein cholesterol (HDL-C) [14,15]. When the six-week intervention finished, considerably (*p* < 0.01) higher TC (3.85 ± 0.09 mM), TG (1.41 ± 0.02 mM), and LDL-C (2.17 ± 0.55 mM) and lower HDL-C (1.41 ± 0.16 mM) contents were found in the serum samples of HLP mice than those of the CK ones (Figure 1C–F). These were in good agreement with the previous findings [1,16]. The animals treated with capsaicin and quercitrin exhibited decreased TC (Figure 1C), TG (Figure 1D), and LDL-C (Figure 1E) and increased HDL-C (Figure 1F) contents compared to the HLP group. Among them, the lowest TC (2.31 ± 0.58 mM), TG (0.67 ± 0.06 mM), and LDL-C (1.14 ± 0.18 mM) values and the highest HDL-C (2.78 ± 0.27 mM) values were found in the Cap+Quer/3:1 group (Figure 1C–F). There has been much evidence supporting the hypolipidemic activity of capsaicin [7]. Yet few data are available describing the effectiveness of quercitrin on the treatment of hyperlipidemia. This research not only provided data to elucidate the positive effect of quercitrin on HLP mice but also clarified the joint role of capsaicin and quercitrin on the amelioration of lipid metabolism disorder.

In addition to the indicators mentioned above, the content of total bile acid (TBA) is an index that is closely related to cholesterol metabolism and can be relied on to assess in vivo lipid levels [17]. A dramatically (*p* < 0.0001) greater TBA level (14.40 ± 0.51 µM) was obtained in the HLP mice than that (4.19 ± 0.29 µM) of the normal control group (Figure 1G). This was consistent with the published results that HLP animals were characterized with increased serum TBA content [18]. Supplementation with capsaicin and quercitrin in the feed made it feasible to alleviate the TBA level in mouse serum, being 8.79 ± 0.66 µM, 6.77 ± 0.39 µM, and 5.54 ± 0.32 µM, which further confirmed the enhanced impact of capsaicin and quercitrin on the mitigation of HLP mice.

Taken together, both capsaicin and quercitrin were effective to modulate hyperlipidemia via down-regulating the TC, TG, LDL-C, and TBA concentrations as well as up-regulating the HDL-C content in mouse serum. A combined effect was noted between capsaicin and quercitrin in relieving hyperlipidemia.

### 3.2. Serum Metabolite Profiles of Mice Exposed to Different Treatments

**Figure 2:**
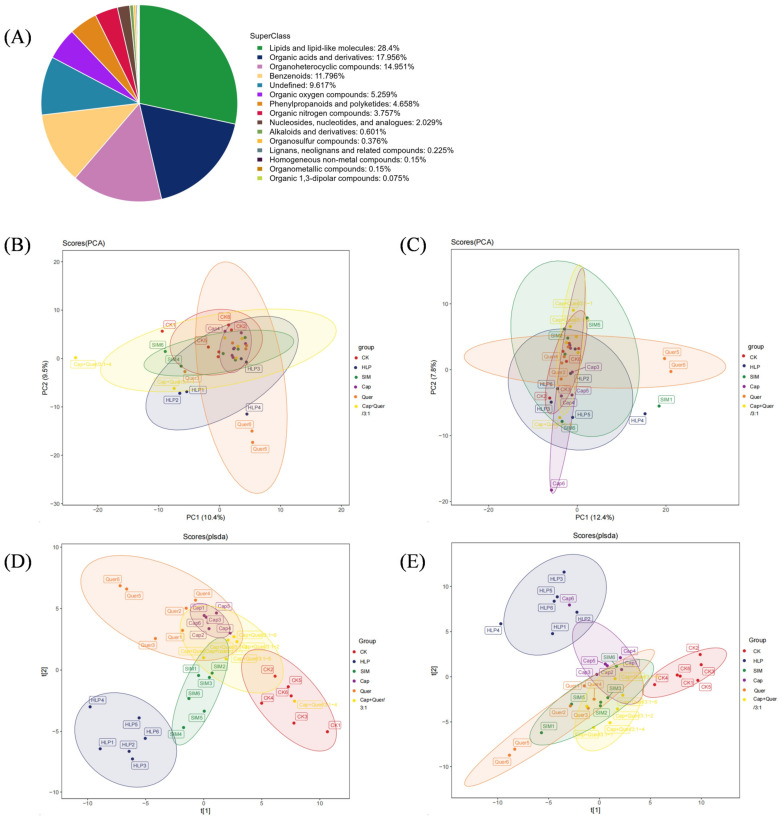
(**A**) The classification and corresponding percentage of all metabolites annotated in the mouse serum samples. PCA score plots of the metabolite data acquired under (**B**) ESI+ mode and (**C**) ESI− mode. PLS-DA score plots of the metabolite data acquired under (**D**) ESI+ mode (R^2^X = 0.62, R^2^Y = 0.99, and Q^2^ = 0.81) and (**E**) ESI− mode (R^2^X = 0.69, R^2^Y = 0.99, and Q^2^ = 0.70). CK, normal control; HLP, hyperlipidemia; SIM, simvastatin; Cap, capsaicin; Quer, quercitrin; COMap+Quer/3:1, capsaicin and quercitrin at a ratio of 3:1.

The metabolite composition of serum collected from all groups of mice was analyzed by using a non-targeted UPLC-MS/MS technique. A number of 9246 and 9394 peak signals were acquired in the serum samples under positive (+) and negative (−) ionization modes, respectively. After alignment with the established database, 776 ESI+ compounds and 555 ESI− compounds were annotated. These metabolites were categorized into 15 super-classes, being 28.40% for lipids and related molecules, 17.96% for organic acids and derivatives, 14.95% for organoheterocyclic compounds, and 11.80% for benzenoids (Figure 2A).

Both univariate and multivariate data statistics were performed on the annotated molecules to illustrate the differences in the metabolite profiles among six groups of mice. Principal component analysis (PCA) found that 19.90% and 20.20% of the total variance between the six animal groups could be explained by the positive (Figure 2B) and negative (Figure 2C) data, respectively. Animals belonging to the CK and HLP groups were clearly separated from the partial least square discriminant analysis (PLS-DA) score plots (Figure 2D,E), demonstrating the obvious divergence in the metabolite composition of these two groups [2,13]. The other four intervened groups were clustered in an overlapped way but clearly separated from the CK and HLP groups (Figure 2D,E), implying that the application of capsaicin and quercitrin caused distinct changes in the metabolite fingerprints of mouse serum [8].

**Figure 3:**
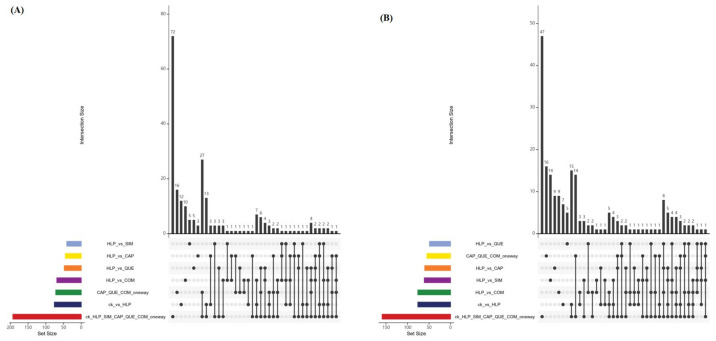
Upset plots of metabolites detected under (**A**) ESI+ mode and (**B**) ESI− mode. The left bar chart displays the total number of variables contained in each original data set, whereas the top bar chart shows the unique and common numbers of each group, representing the number of intersection variables in the intersection situation through vertical correspondence. The intersection point in the lower right corner demonstrates the corresponding data set indicated on the left side through horizontal correspondence. By vertically connecting points, it represents the presence of intersection between the corresponding datasets.

Figure 3A,B display the upset plots of the metabolites present in the six groups of mouse serum. As for those detected under the positive ESI mode, 72 metabolites were commonly present in all groups of serum samples (Figure 3A), while for the negative ESI mode, 47 molecules were simultaneously detected (Figure 3B). The importance of these compounds for the separation of the six groups of serum samples were evaluated based on their corresponding *p* value and VIP score. The detailed information of the top 20 most significantly differential metabolites is summarized in Table 1. The serum samples collected from the normal control group contained the highest abundance of nine components, namely, 3-hydroxyxanthone, propranolol, 2-amino-3,8-dimethylimidazo[4,5-f]quinoxaline, 1-(1z-hexadecenyl)-sn-glycero-3-phosphocholine, oxybutynin, 14-hydroxy-4z,7z,10z,12e,16z,19z-docosahexaenoic acid, 2,6′-dimethoxy-2′-hydroxychalcone, 12-oxo-5z,8z,10e,14z-eicosatetraenoic acid, and *N*-acetylhistidine (Table 1). Most of them were lipid and lipid-derived molecules and strongly associated with the in vivo lipid metabolism of animals [19]. More importantly, there were long-chain polyunsaturated fatty acids, which have been widely recognized for their multiple health advantages, such as the prevention of cardiovascular disease and atherosclerosis [20,21]. A downward trend in the relative abundance of these ingredients was shown in the HLP group (Table 1). When supplied with capsaicin and quercitrin, changes were observed in the intensity of these compounds compared to the HLP group (Table 1), indicating the effectiveness of these two bio-active compounds. On the other hand, the mice with hyperlipidemia contained significantly higher levels of o-succinyl-l-homoserine, L-citrulline, pseudouridine, (4e,8e)-10-(4-hydroxy-6-methoxy-7-methyl-3-oxo-1h-2-benzofuran-5-yl)-4,8-dimethyldeca-4,8-dienoic acid, daidzein 4′-sulfate, γ-glutamylleucine, and 5-methoxyindoleacetate (Table 1). Most of them were organic acid and related compounds, which can be metabolized into various amines [22]. There have been many publications confirming the negative roles of amine compounds like disrupting cholesterol metabolism, the incidence of thrombosis and atherosclerosis, increasing the risk of nonalcoholic fatty liver, etc. [23,24]. These data imply the occurrence of lipid metabolism disorder might have a connection with the production of amines via organic acid metabolism. Supplementation with both capsaicin and quercitrin can alleviate the levels of organic acids present in the mouse serum. In addition to all mentioned above, propentofylline was another critical differential compound, which was present at the highest abundance in the Quer group (Table 1). It was beneficial for insulin resistance, diabetes, cardiovascular disease, and atherosclerosis via elevating the levels of acetal phospholipids and sphingomyelin [25].

**Figure 4:**
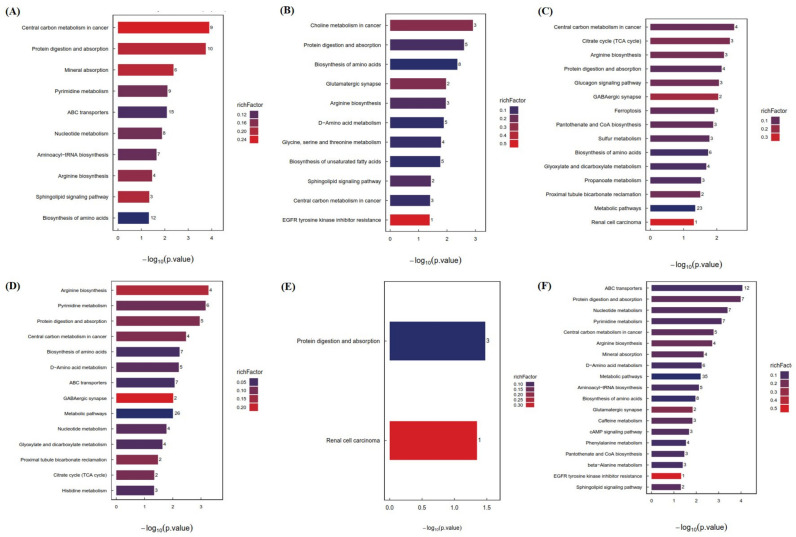
The top enriched KEGG pathways of significantly differential metabolites between (**A**) the six groups of mouse serum, (**B**) CK vs. HLP, (**C**) HLP vs. SIM, (**D**) HLP vs. Cap, (**E**) HLP vs. Quer, and (**F**) HLP vs. Cap+Quer/3:1.

The most significantly metabolic pathways participated by these differential molecules are summarized in Figure 4A–F. When considering the six groups together, the top 10 enriched KEGG pathways were central carbon metabolism in cancer, protein digestion and absorption, mineral absorption, pyrimidine metabolism, ABC transporters, nucleotide metabolism, aminoacyl-tRNA biosynthesis, arginine biosynthesis, the sphingolipid signaling pathway, and the biosynthesis of amino acids (Figure 4A). Most of them have been involved in previous publications for their correlation with lipid metabolism [13,26,27]. For example, protein digestion and absorption can affect the release of hormones that regulate the digestibility and anabolism of fats [26]. Pyrimidine metabolism was closely related to mitochondrial activity, which has been proved to be associated with obesity and type 2 diabetes [28]. ABC transporters cover a heterogeneous group of ATP-dependent transporters that promote the physiological processes of various substrates in animal tissues, including lipids [29]. Arginine metabolism is an important pathway for regulating liver injury caused by lipid metabolism disorders [30]. Mice fed with a high-fat diet have the possibility to accumulate lipids in their liver tissues. The accumulated triacylglycerols can be esterified into ceramides through the sphingolipid pathway, leading to increased obesity [31].

As for the CK vs. HLP groups, most of the top KEGG pathways were similar to those of the six groups (Figure 4B). One exception was EGFR tyrosine kinase inhibitor resistance, which was also observed in the comparison of HLP vs. Cap+Quer/3:1 (Figure 4F). This pathway is related to lipid metabolism via regulating the expression of EGFR protein. There were 15 and 14 KEGG pathways enriched in the comparison of HLP vs. SIM (Figure 4C) and HLP vs. Cap (Figure 4D), respectively. The citrate cycle (TCA cycle) was commonly present in these two comparisons (Figure 4C,D), which worked to combine carbohydrate, lipid, and protein metabolism [32]. A reduction in TCA cycle intermediates could lead to increased glycolysis and a flux in citric acid for fatty acid and lipid synthesis [32]. Unlike the other comparisons, there were only two pathways (protein digestion and absorption as well as renal cell carcinoma) enriched between the HLP and Quer groups (Figure 4E). Renal cell carcinoma was also present in Figure 4C. Renal cell carcinoma primarily occurred in the renal tubular epithelial cells, which are vulnerable to the imbalance of lipid absorption and application, further exacerbating lipid accumulation and lipotoxicity [33].

The above data indicate that the occurrence of lipid metabolism disorders involves multiple signaling pathways, seen from CK vs. HLP. Capsaicin and quercitrin, either alone or in combination, affected lipid metabolism disorders through different pathways. Thus, we consider this as a systematic network which needs much further research in the future to clarify the interactions among these signaling pathways.

### 3.3. Variations in Relative Expression Levels of Key Proteins in Mouse Liver Exposed to Different Treatments

**Figure 5:**
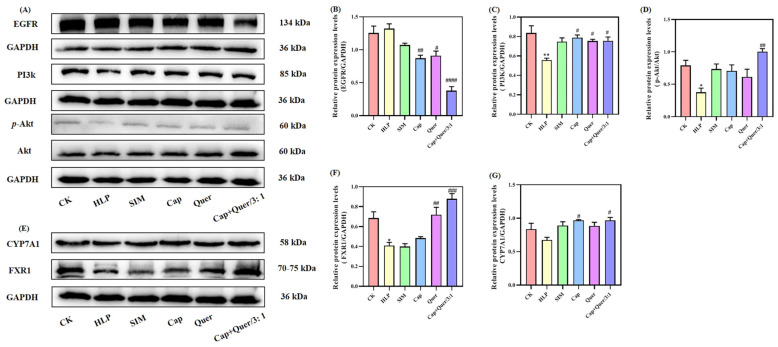
The effect of capsaicin, quercitrin, and their combination on the (**A**) intensity of immunoblot bands, the relative protein expression levels of (**B**) epidermal growth factor receptor (EGFR), (**C**) PI3K, (**D**) p-Akt/Akt, (**E**) intensity of immunoblot bands, (**F**) farnesoid X receptor 1 (FXR1), and (**G**) cholesterol 7α-hydroxylase (CYP7A1). Compared to the control group, * *p* < 0.05 and ** *p* < 0.01. Compared to the hyperlipidemic group, # *p* < 0.05, ## *p* < 0.01, ### *p* < 0.001, and #### *p* < 0.0001.

Lipid metabolism involves multiple organs, like the liver and gall bladder, and is regulated by a complicated interaction of various signaling pathways [34,35]. In the current study, the degrees of expression of epidermal growth factor receptor (EGFR), PI3K, p-Akt, and Akt in the liver tissues were examined (Figure 5A). EGFR, belonging to the receptor tyrosine kinase (RTK) family, is positively related to the occurrence of hepatic steatosis, cholesterol synthesis, and upward intrahepatic lipid content [34,35,36]. Figure 5B illustrates the relative expression data of EGFR. A rising tendency in the relative expression level of EGFR was observed in the HLP group (1.32 ± 0.12) compared to the CK group (1.25 ± 0.18). After six-week intervention with capsaicin and quercitrin, the expression of EGFR significantly (*p* < 0.05) declined, especially in the Cap+Quer/3:1 group (Figure 5B). These findings imply that the hypolipidemic bioactivity of capsaicin and quercitrin might be related to the down-regulation of EGFR expression.

In addition to EGFR, the PI3K/Akt signaling pathway can target the liver organ to maintain lipid metabolism homeostasis via regulating cholesterol synthesis and transformation [37]. Intragastric administration with capsaicin and quercitrin in the mice feed enhanced the expression amount of PI3K (Figure 5C) and p-Akt/Akt (Figure 5D). The activation of the PI3K/Akt signaling pathway is able to improve glucose and lipid metabolism [37]. Thus, the positive role of capsaicin and quercitrin could plausibly contribute to the initiation of the PI3K/Akt signaling pathway.

The liver is a primary organ for cholesterol synthesis and degradation into bile acids [38]. Figure 5E shows the relative expression levels of farnesoid X receptor 1 (FXR1) and cholesterol 7α-hydroxylase (CYP7A1). CYP7A1 is a critical enzyme catalyzing the production of bile acids from cholesterol [32], whilst the nuclear receptor FXR1 works to regulate the activity of CYP7A1 and alleviate the accumulation of fatty acids in the liver [39,40]. Both FXR1 and CYP7A1 exhibited downward expression levels in the HLP animals in contrast to the CK group (Figure 5F,G). This was in alignment with previous evidence that the expression of FXR and CYP7A1 in the liver tissues of hyperlipidemic mice was down-regulated [41,42]. Both capsaicin and quercitrin promoted the expression of FXR1 (Figure 5F) and CYP7A1 (Figure 5G) in the liver tissues, indicating that capsaicin and quercitrin intervention effectively accelerated the metabolism of cholesterol into bile acids, thereby reducing cholesterol accumulation.

## 4. Conclusions

The current study provides evidence for the combined role of capsaicin and quercitrin on the improvement of hyperlipidemia via lowering the levels of fasting glucose, total cholesterol, total triglycerides, total bile acids, and low-density lipoprotein cholesterol as well as lifting the content of high-density lipoprotein cholesterol. The underlying mechanism was explored by an evaluation on the metabolite profiles of mouse serum. Obvious separations were observed between the normal control, hyperlipidemic, and administration groups. The bio-informatics data demonstrate that the positive effect of capsaicin and quercitrin on maintaining lipid homeostasis is related to both lipid and amino acid metabolism pathways. The key signaling proteins were targeted on the EGFR/PI3K/Akt in this study. Capsaicin and quercitrin also enhanced the expression of CYP7A1 and FXR1 to accelerate the lipid metabolism of hyperlipidemic mice.

## Figures and Tables

**Table 1:** The top 20 most significantly differential metabolites among the six groups of mouse serum.

No.	Metabolites	Classes	Average Abundance						*p* Value	VIP Score
			CK	HLP	SIM	Cap	Quer	Cap+Quer/3:1		
1	1-Hexadecyl-2-(5z,8z,11z,14z-eicosatetraenoyl)-sn-glycero-3-phosphocholine	Lipids and lipid-like molecules	1.10 × 10^8^	1.04 × 10^8^	9.62 × 10^7^	9.22 × 10^7^	1.30 × 10^8^	9.32 × 10^7^	1.06 × 10^−4^	1.51
2	N-oleoyl-d-erythro-sphingosylphosphorylcholine	Lipids and lipid-like molecules	8.15 × 10^7^	1.24 × 10^8^	9.83 × 10^7^	1.02 × 10^8^	9.78 × 10^7^	1.13 × 10^8^	1.19 × 10^−4^	1.40
3	3-Hydroxyxanthone	Organoheterocyclic compounds	1.09 × 10^7^	5.41 × 10^6^	1.00 × 10^7^	9.15 × 10^6^	7.97 × 10^6^	7.81 × 10^6^	1.35 × 10^−4^	1.38
4	Propranolol	N.A.	2.34 × 10^8^	2.10 × 10^8^	1.77 × 10^8^	1.81 × 10^8^	1.36 × 10^8^	1.63 × 10^8^	1.35 × 10^−4^	1.23
5	2-Amino-3,8-dimethylimidazo[4,5-f]quinoxaline	Organoheterocyclic compounds	6.21 × 10^6^	5.78 × 10^6^	5.00 × 10^6^	4.94 × 10^6^	3.59 × 10^6^	4.05 × 10^6^	1.53 × 10^−4^	1.22
6	1-(1z-Hexadecenyl)-sn-glycero-3-phosphocholine	Lipids and lipid-like molecules	8.21 × 10^7^	6.51 × 10^7^	6.74 × 10^7^	6.27 × 10^7^	6.48 × 10^7^	6.35 × 10^7^	2.00 × 10^−4^	1.27
7	Oxybutynin	N.A.	1.20 × 10^7^	9.21 × 10^6^	8.52 × 10^6^	8.39 × 10^6^	6.30 × 10^6^	6.74 × 10^6^	2.06 × 10^−4^	1.23
8	O-succinyl-l-homoserine	Lipids and lipid-like molecules	1.11 × 10^7^	1.69 × 10^7^	1.05 × 10^7^	1.27 × 10^7^	1.33 × 10^7^	1.01 × 10^7^	2.22 × 10^−4^	1.36
9	L-citrulline	Phenylpropanoids and polyketides	5.27 × 10^7^	7.31 × 10^7^	4.85 × 10^7^	5.08 × 10^7^	5.70 × 10^7^	4.80 × 10^7^	2.61 × 10^−4^	1.28
10	Propentofylline	Lipids and lipid-like molecules	8.27 × 10^6^	7.89 × 10^6^	7.05 × 10^6^	6.19 × 10^6^	1.08 × 10^7^	1.05 × 10^7^	2.61 × 10^−4^	1.54
11	Pseudouridine	Organic acids and derivatives	1.09 × 10^8^	1.27 × 10^8^	1.11 × 10^8^	1.10 × 10^8^	9.26 × 10^7^	1.05 × 10^8^	1.09 × 10^−4^	1.25
12	Cefaclor	Organoheterocyclic compounds	9.40 × 10^6^	6.82 × 10^6^	1.03 × 10^7^	3.83 × 10^6^	8.62 × 10^6^	4.31 × 10^6^	1.19 × 10^−4^	1.59
13	14-Hydroxy-4z,7z,10z,12e,16z,19z-docosahexaenoic acid	Organoheterocyclic compounds	2.23 × 10^8^	1.05 × 10^8^	1.82 × 10^8^	1.36 × 10^8^	7.03 × 10^7^	9.81 × 10^7^	1.61 × 10^−4^	1.19
14	(4e,8e)-10-(4-Hydroxy-6-methoxy-7-methyl-3-oxo-1h-2-benzofuran-5-yl)-4,8-dimethyldeca-4,8-dienoic acid	Organic acids and derivatives	3.49 × 10^6^	1.05 × 10^7^	2.81 × 10^6^	2.79 × 10^6^	6.01 × 10^6^	3.98 × 10^6^	1.74 × 10^−4^	1.25
15	Daidzein 4′-sulfate	Organic acids and derivatives	2.47 × 10^6^	1.09 × 10^7^	5.95 × 10^6^	1.27 × 10^6^	4.65 × 10^6^	1.24 × 10^6^	1.79 × 10^−4^	1.20
16	2,6′-Dimethoxy-2′-hydroxychalcone	Nucleosides, nucleotides, and analogues	7.53 × 10^6^	6.47 × 10^6^	4.92 × 10^6^	4.55 × 10^6^	4.39 × 10^6^	3.77 × 10^6^	2.41 × 10^−4^	1.20
17	Gamma-glutamylleucine	Lipids and lipid-like molecules	3.94 × 10^6^	4.80 × 10^6^	3.17 × 10^6^	3.30 × 10^6^	2.79 × 10^6^	2.72 × 10^6^	2.97 × 10^−4^	1.19
18	12-oxo-5z,8z,10e,14z-Eicosatetraenoic acid	Phenylpropanoids and polyketides	1.13 × 10^8^	5.09 × 10^7^	8.28 × 10^7^	6.25 × 10^7^	3.36 × 10^7^	4.24 × 10^7^	3.00 × 10^−4^	1.15
19	N-acetylhistidine	Organic acids and derivatives	1.44 × 10^8^	1.03 × 10^8^	9.05 × 10^7^	1.01 × 10^8^	9.11 × 10^7^	9.46 × 10^7^	3.02 × 10^−4^	1.20
20	5-Methoxyindoleacetate	Organoheterocyclic compounds	5.78 × 10^7^	7.66 × 10^7^	5.05 × 10^7^	4.86 × 10^7^	2.15 × 10^7^	3.32 × 10^7^	4.11 × 10^−4^	1.16

Note: CK, normal control; HLP, hyperlipidemia; SIM, simvastatin; Cap, capsaicin; Quer, quercitrin; Cap+Quer/3:1, capsaicin and quercitrin at a ratio of 3:1; VIP, variable importance in projection; N.A., not applicable.

## Data Availability

The original contributions presented in the study are included in the article and Supplementary Materials, further inquiries can be directed to the corresponding author.

## Supplementary Materials

The following supporting information can be downloaded at: https://www.mdpi.com/article/10.3390/foods13233727/s1, Figure S1: Molecular structures of capsaicin and quercitrin; Figure S2: Images of animals under different treatments.
