# Supplementary figures and images for: Capsaicin and Quercitrin Maintained Lipid Homeostasis of Hyperlipidemic Mice: Serum Metabolomics and Signaling Pathways
Source: Foods. 2024 Nov 21;13(23):3727. doi: 10.3390/foods13233727 (PMC11640311; doi:10.3390/foods13233727)

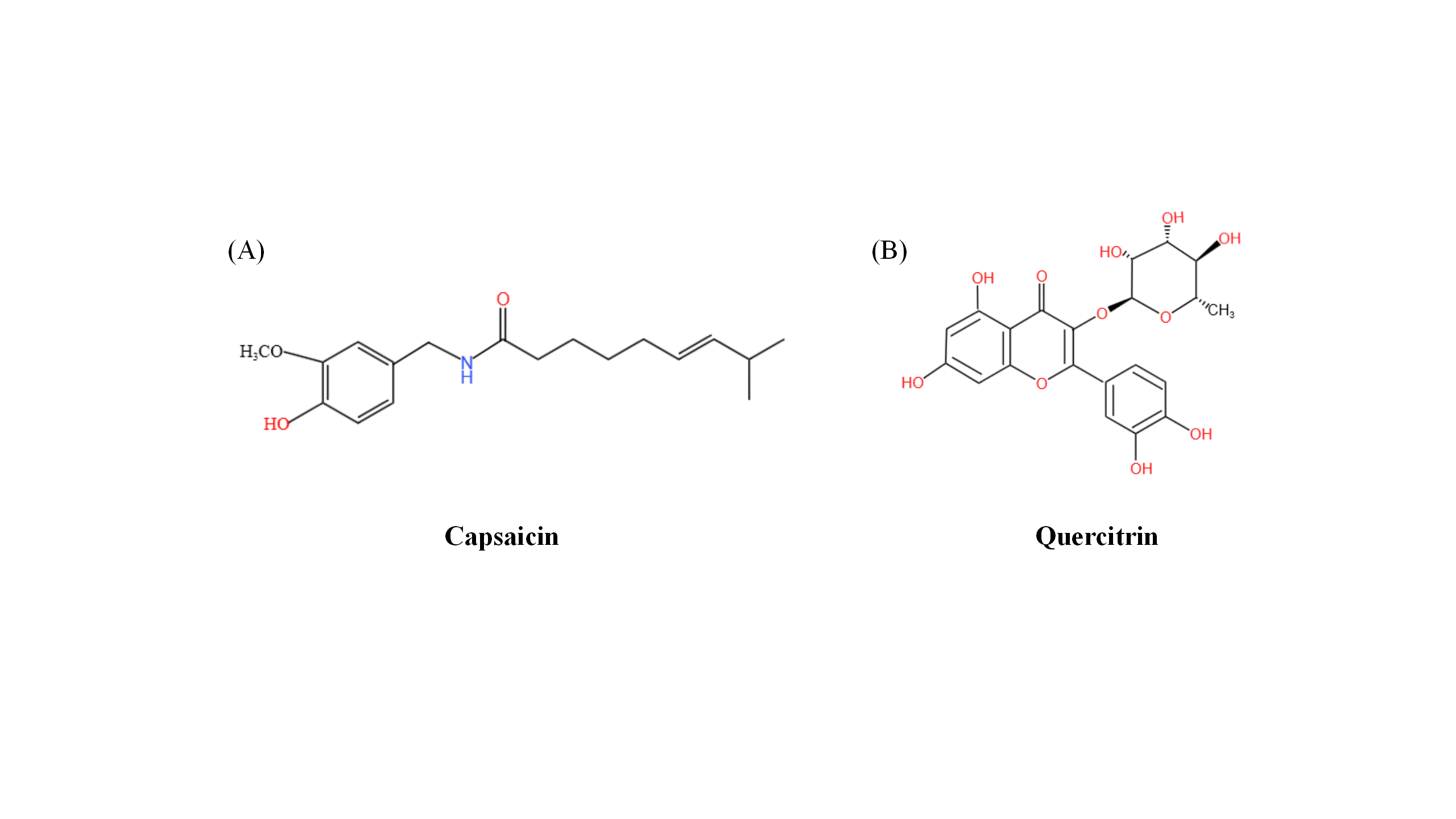

Supplement: Supplementary file 1 [file foods-13-03727-s001.zip › Supplementary Figure 1.png]

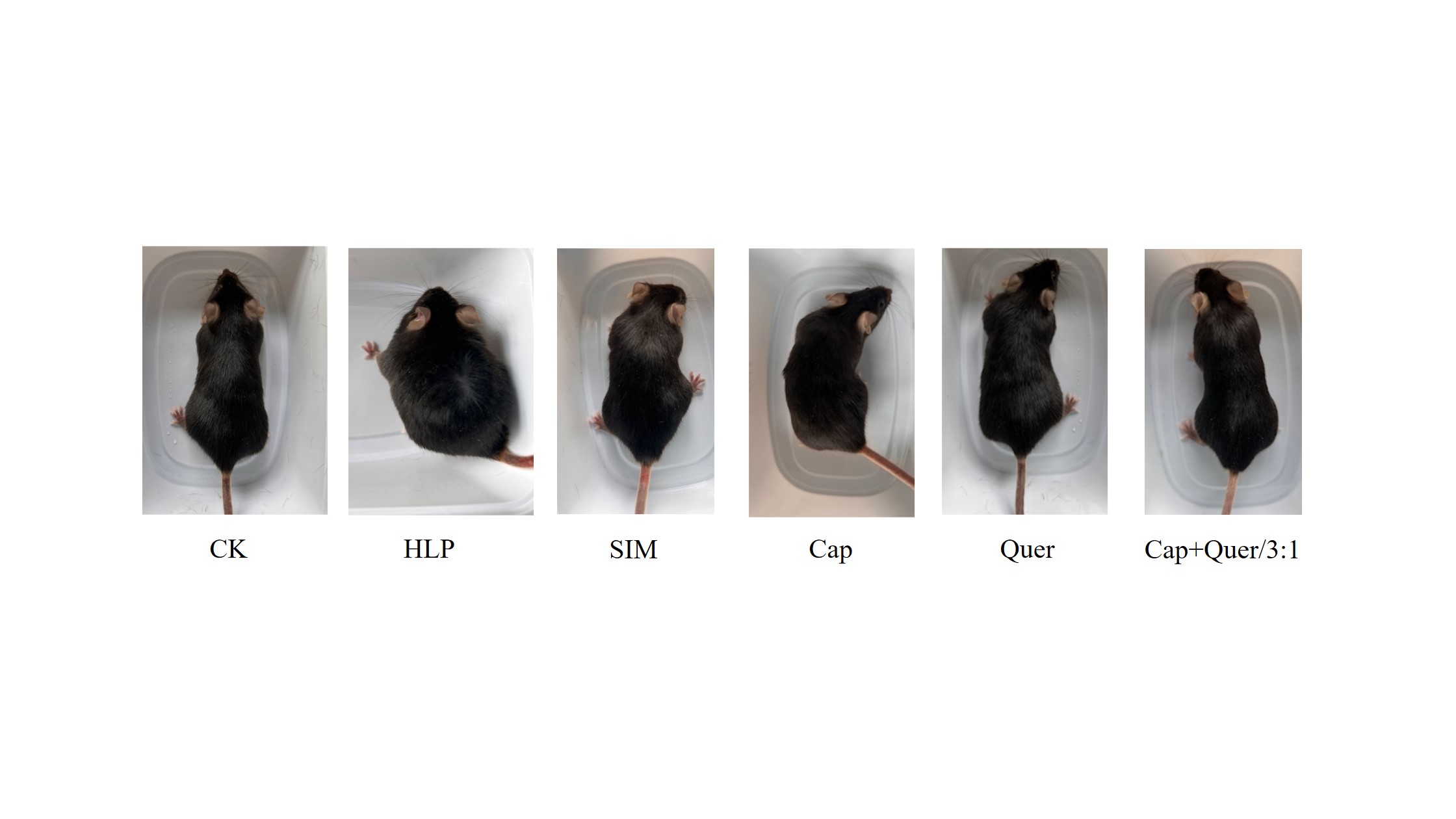

Supplement: Supplementary file 1 [file foods-13-03727-s001.zip › Supplementary Figure 2.jpg]
